# A robust bulk-solvent correction and anisotropic scaling procedure

**DOI:** 10.1107/S0907444905007894

**Published:** 2005-06-24

**Authors:** Pavel V. Afonine, Ralf W. Grosse-Kunstleve, Paul D. Adams

**Affiliations:** a Lawrence Berkeley National Laboratory, One Cyclotron Road, Building 64R0121, Berkeley, CA 94720, USA

**Keywords:** bulk-solvent correction, anisotropic scaling

## Abstract

A reliable method for the determination of bulk-solvent model parameters and an overall anisotropic scale factor is of increasing importance as structure determination becomes more automated. Current protocols require the manual inspection of refinement results in order to detect errors in the calculation of these parameters. Here, a robust method for determining bulk-solvent and anisotropic scaling parameters in macromolecular refinement is described. The implementation of a maximum-likelihood target function for determining the same parameters is also discussed. The formulas and corresponding derivatives of the likelihood function with respect to the solvent parameters and the components of anisotropic scale matrix are presented. These algorithms are implemented in the *CCTBX* bulk-solvent correction and scaling module.

## Abstract 2

A robust method for determining bulk-solvent and anisotropic scaling parameters for macromolecular refinement is described. A maximum-likelihood target function for determination of flat bulk-solvent model parameters and overall anisotropic scale factor is also proposed.

## 1. Introduction

Analysis of the Protein Data Bank (PDB; Bernstein *et al.*, 1977 ▶; Berman *et al.*, 2000 ▶) shows that macromolecular crystals contain a significant amount of disordered solvent. The total solvent content varies around a mean of 55%, with a lower bound of approximately 20% and an upper bound of approximately 95%. The contribution of this bulk solvent to the diffracted amplitudes becomes non-negligible at lower resolution (*d* > 8.0 Å). In the past, it has been common practice to truncate the low-resolution data and use only middle- and high-resolution shells for crystallographic calculations. More recently, it has been demonstrated that low-resolution data are very important for electron-density map analysis (Urzhumtsev, 1991 ▶), crystallographic refinement (Kostrewa, 1997 ▶) and the translation search in the molecular-replacement method (Urzhumtsev & Podjarny, 1995 ▶; Fokine & Urzhumtsev, 2002*b* ▶). For a review and more complete set of references see, for example, Jiang & Brünger (1994 ▶), Badger (1997 ▶) and Urzhumtsev (2000 ▶).

Jiang & Brünger (1994 ▶) demonstrated that a flat bulk-solvent model (Phillips, 1980 ▶) is the most reliable model and proposed an algorithm for calculation of the parameters. This involves the calculation of a solvent mask and the determination of two bulk-solvent parameters, *k*_sol_ and *B*_sol_. Fokine & Urzhumtsev (2002*a* ▶) analyzed the distribution of bulk-solvent parameters and provided a more physical insight for this model. Alternatively, an exponential model for correcting for the effects of bulk solvent (Moews & Kretsinger, 1975 ▶; Tronrud, 1997 ▶) can be used. This is available in some refinement programs: *SHELX* (Sheldrick & Schneider, 1997 ▶), *REFMAC* (Murshudov *et al.*, 1997 ▶; *REFMAC* also provides the option for the flat bulk solvent described above) and *TNT* (Tronrud, 1997 ▶). However, it has been shown that this method is only correct at very low resolution (lower than 15 Å) and inappropriate at higher resolution (Podjarny & Urzhumtsev, 1997 ▶). Therefore, in this work we only consider the flat bulk-solvent model.

The bulk-solvent parameters *k*_sol_ and *B*_sol_ are usually determined along with an overall scale factor between observed and calculated structure factors. It was demonstrated that the use of an anisotropic overall scale factor is physically more appropriate and can significantly reduce both the *R* and *R*_free_ factors (Sheriff & Hendrickson, 1987 ▶; Murshudov *et al.*, 1998 ▶). The criterion traditionally used to attain this goal is
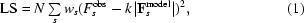
where *N* = 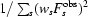 is a normalization factor (Brünger *et al.*, 1989 ▶; Jiang & Brünger, 1994 ▶), the model structure factors
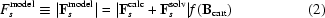
accumulate structure factors from the atomic model **F** ^calc^ (macromolecule plus ordered solvent), contribution from the bulk solvent
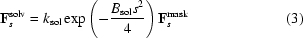
and overall anisotropic scale factor can be either in exponential form (Sheriff & Hendrickson, 1987 ▶) with six parameters to be determined, as implemented in *CNS* (Brünger *et al.*, 1998 ▶) and *REFMAC* (Murshudov *et al.*, 1998 ▶),
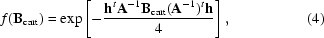
or the linear function of 12 parameters as implemented in *SHELXL* (Usón *et al.*, 1999 ▶; Parkin *et al.*, 1995 ▶). In this work, we consider only the exponential form of the anisotropic scale factor (4).

The scale *k* is chosen such that the derivative of LS with respect to *k* is zero, *k* = 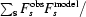 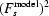, which is a necessary condition to make LS minimal (Brünger *et al.*, 1989 ▶), **h** is a column vector with the Miller indices of a reflection, **h** ^*t*^ is the transposed vector, **B**_cart_, the overall anisotropic scale matrix, has the same units and conversion rules as **B**_cart_ defined in equations (2), (3*b*) and (7) of Grosse-Kunstleve & Adams (2002 ▶), **A** is an orthogonalization matrix, *k*_sol_ and *B*_sol_ are the flat bulk-solvent model parameters, *s* ^2^ = **h** ^*t*^ G*h, where G* is the reciprocal-space metric tensor, and **F** ^mask^ are the structure factors calculated from a molecular mask (a binary function with zero values in the protein region and unit values in the solvent region). The use of **B**_cart_ makes it straightforward to apply the isotropic component of the tensor to both *B*_sol_ and the atomic isotropic *B* factors in order to compensate for the high correlation of these parameters with the overall anisotropic scale matrix.

The correction for bulk solvent and scaling is usually the first step in a crystallographic refinement protocol. If a least-squares-based refinement procedure is chosen, where a target function of form (1) is used in optimization of atomic model parameters, then the use of the same target function for the scaling and bulk-solvent parameters determination is well justified. However, if the maximum-likelihood-based refinement strategy is chosen (Bricogne, 1991 ▶; Pannu & Read, 1996 ▶; Bricogne & Irwin, 1996 ▶; Murshudov *et al.*, 1997 ▶), the use of function (1) for bulk-solvent and scale-parameter determination is less justified. In this case, it is more natural to also determine the bulk-solvent and anisotropic scale parameters from the likelihood function, allowing all the parameters to be optimized using the same criterion. The use of a likelihood function for the determination of bulk-solvent parameters has been discussed by Blanc *et al.* (2004 ▶).

It has been observed that the determination of bulk-solvent parameters is a numerically challenging problem (Jiang & Brünger, 1994 ▶; Fokine & Urzhumtsev, 2002*a* ▶). Inclusion of the anisotropic overall scale factor makes the problem even more complicated. Some possible reasons for this are the following.

(i) The quality and/or completeness of the low-resolution diffraction data may be insufficient.
(ii) The starting values for *k*_sol_ and *B*_sol_ may be far from the correct values.
(iii) The parameters *k*_sol_, *B*_sol_, *k* and **B**_cart_ are highly correlated. This may result in instability of the minimization procedure.
(iv) Optimization of a function of two exponentials is generally a non-trivial problem.

Therefore, it is not surprising to find 95 models in the PDB (see selection criteria below; scoring performed August 2004) with bulk-solvent parameters beyond the physically meaningful range discussed in Fokine & Urzhumtsev (2002*a* ▶).

In this paper, we describe a robust protocol for the determination of bulk-solvent and anisotropic scaling parameters using both maximum-likelihood and least-squares target functions and its implementation in the *Computational Crystallographic Toolbox* (*CCTBX*; Grosse-Kunstleve *et al.*, 2002 ▶).

## 2. The maximum-likelihood target function and its derivatives with respect to bulk-solvent parameters and components of the anisotropic scale matrix

The negative logarithm of the maximum-likelihood function (Lunin & Skovoroda, 1995 ▶), which is implemented in *CCTBX* as one of the crystallographic target functions for structure refinement, can be presented as
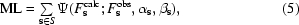
with
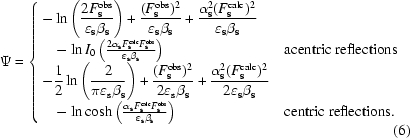
Here, 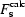 is the calculated structure-factor magnitude for the reflection **s** from the available atomic model. The coefficient ∊**_s_** depends on the three-dimensional index **s** and on the space group and is equal to the number of symmetry operations that, when applied to the vector **s**, leave it unchanged. The parameters α**_s_** and β**_s_** accumulate the uncertainties in atomic coordinates and temperature factors (Lunin & Urzhumtsev, 1984 ▶; Read, 1986 ▶, 1990 ▶, 2001 ▶; Lunin & Skovoroda, 1995 ▶; Pannu & Read, 1996 ▶; Urzhumtsev *et al.*, 1996 ▶). It is worth noting that the scale coefficient between observed and calculated structure factors, if not introduced explicitly, is also accumulated in these two parameters.

The explicit introduction of the anisotropic scale factor and the contribution from the bulk solvent into (5) can be realised by replacing 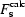 with 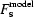 as defined in (2), 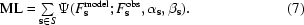
The derivatives of Ψ with respect to the six anisotropic scale-matrix elements **B**_cart_ and the solvent parameters *k*_sol_ and *B*_sol_ required for first-derivative minimization methods such as *LBFGS* (Liu & Nocedal, 1989 ▶) are provided in Appendix *A* [App appa].

## 3. Algorithm for determination of *k*_sol_, *B*_sol_ and **B**_cart_

Fokine & Urzhumtsev (2002*a* ▶) have shown that the bulk-solvent parameters *k*_sol_ and *B*_sol_ are distributed around 0.35 e Å^−3^ and 46 Å^2^ and the physically reasonable range for these parameters can be approximately defined as *k*_sol_ ∈ (0.1, 0.8) and *B*_sol_ ∈ (10, 80). These observations make it possible to implement a systematic search procedure for the determination of *k*_sol_ and *B*_sol_, therefore making the whole protocol very robust and insensitive to the potential minimization problems mentioned above.

**Figure 1:**
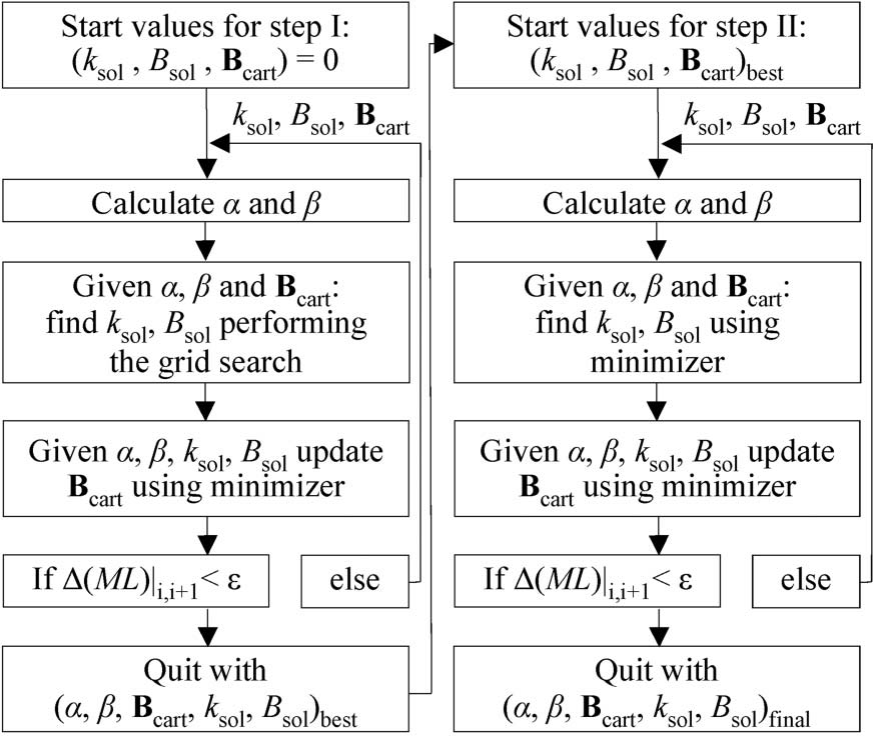
Algorithm for calculation of flat bulk-solvent model parameters *k*_sol_ and *B*_sol_ and the anisotropic scale matrix **B**_cart_ as implemented in *CCTBX*.

Fig. 1 ▶ outlines the algorithm implemented in the *CCTBX* using the likelihood function. Starting from zero values for *k*_sol_, *B*_sol_ and **B**_cart_, the values for α and β (Lunin & Skovoroda, 1995 ▶) are calculated using cross-validation data with smoothing over resolution shells using spline functions (Lunin & Skovoroda, 1997 ▶). The value of the ML function (7) is evaluated at this initial point. In the next step, a grid-search procedure is applied in order to find *k*_sol_ and *B*_sol_: for each trial pair (*k*_sol_, *B*_sol_) the parameters α, β are updated and the value of ML is recalculated. The set of (α, β, *k*_sol_, *B*_sol_) with the minimum value of the function ML is then selected. The *LBFGS* minimization algorithm is used to optimize ML with respect to the six components of the **B**_cart_ tensor with the parameters for α, β, *k*_sol_ and *B*_sol_ found in the previous step held constant. Symmetry restrictions are applied to the elements of **B**_cart_ (Sheriff & Hendrickson, 1987 ▶); however, they can optionally be turned off. The value of the ML function is evaluated again in order to determine if the procedure has converged; convergence has taken place when the difference of the target function between two steps is less then a certain tolerance value. This tolerance value is fixed as 1% of the relative drop in the target function value. Otherwise, the procedure is repeated starting with the set of parameters obtained in the previous step until convergence is reached.

For reasons of efficiency, the sampling step used in the grid-search procedure is quite coarse. For example, *B*_sol_ is by default varied within the range 10–80 Å^2^ with a sampling step of 5 Å^2^. Finer sampling can be used, but increases the computational time. The parameters *k*_sol_ and *B*_sol_ obtained in such a way are then used as the start values for the next calculations, which are the same as above but with the grid search for *k*_sol_ and *B*_sol_ replaced with the *LBFGS* minimization. This allows *k*_sol_ and *B*_sol_ to be determined more precisely. However, if the minimization fails the best parameters from the previous step are retained. The procedure using the LS function (1) as a criterion is implemented in a similar way. The default parameters for the mask calculation are *r*_solv_ = 1.0 Å and *r*_shrink_ = 1.0 Å and the grid step is the highest resolution of the data divided by 4 (for the definition of these parameters, see Jiang & Brünger, 1994 ▶).

It should be emphasized that all available data are used throughout the procedure without any partitioning by resolution.

## 4. Numerical tests

The goal of this test was to compare the performance of two proposed algorithms with least-squares (1) and maximum-likelihood (7) target functions using simulated models of different quality with simulated experimental data.

We used the model of a Fab fragment of a monoclonal antibody (Fokine *et al.*, 2000 ▶) which consists of 439 amino acid residues and 213 water molecules. The crystals belong to space group *P*2_1_2_1_2_1_, with unit-cell parameters *a* = 72.24, *b* = 72.01, *c* = 86.99 Å. The values of 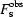 were simulated by the amplitudes of structure factors calculated from the complete exact model at 2.2 Å resolution. The contributions of bulk solvent with *k*_sol_ = 0.25 e Å^−3^ and *B*_sol_ = 55.0 Å^2^ and anisotropy with the diagonal elements (4, 8, −6) Å^2^ were added to 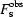 in accordance with (2) and (3). Random errors with mean values in the range 0.0–0.6 Å were then introduced into the atomic coordinates of the complete exact model. Incomplete models were obtained by random deletion of 5 and 10% of atoms from the ensemble of models with errors; this generated a total of 21 models.

**Figure 2:**
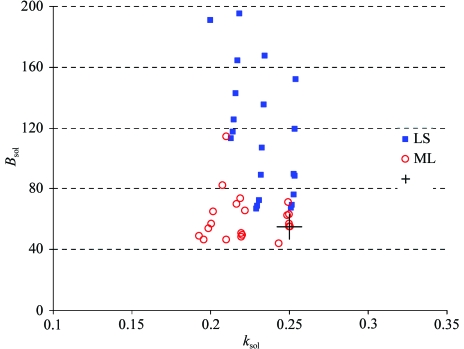
Flat bulk-solvent model parameters *k*_sol_ and *B*_sol_ determined for 21 test models (see text for details of the models) using the least-squares (LS) or maximum-likelihood (ML) target functions.

Fig. 2 ▶ shows the distribution of bulk-solvent parameters obtained using (1) and (7) as the target functions. With the exception of two pairs, all pairs of *k*_sol_ and *B*_sol_ obtained with the likelihood target are within the physically reasonable range and, depending on the model quality, relatively close to the exact value of 0.25 e Å^−3^ and 55.0 Å^2^. In contrast, most of the solvent parameters calculated using the least-squares function are outside the correct range, with some values for *B*_sol_ reaching 200 Å^2^. This is not unexpected as the least-squares target does not include any mechanism to correct for model incompleteness and hence all eight adjustable parameters, *k*_sol_, *B*_sol_ and **B**_cart_, model the contribution from bulk solvent and anisotropy along with the model errors and incompleteness. For the likelihood-based refinement the distribution parameters α and β compensate for model errors and incompleteness. It is the high correlation between all of the model parameters which makes it necessary to develop the thorough and robust algorithm described in the previous section.

## 5. Tests with experimental data

In order to evaluate this new procedure for bulk-solvent correction and anisotropic scaling, we selected all ‘problem’ models from the PDB, *i.e.* those with physically unreasonable values for the flat bulk-solvent model parameters. The exact selection criteria were structures solved by X-ray diffraction with the flat bulk-solvent model used, *k*_sol_ < 0.1 or *k*_sol_ > 1.0 e Å^−3^ and *B*_sol_ < 10 or *B*_sol_ > 100 Å^2^. This selected 95 models. The further demand for experimental data and cross-validation flags (‘test’ set of reflections) combined with an evaluation of the overall data correctness reduced the selected number of models to 35.

**Figure 3:**
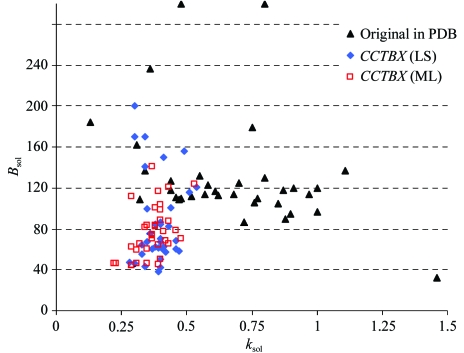
Flat bulk-solvent model parameters *k*_sol_ and *B*_sol_ for 35 structures selected from the PDB (PDB codes 1ci3, 1gzk, 1jh7, 1jj1, 1jvx, 1jzb, 1ev8, 1evf, 1k33, 1ijk, 1izr, 1kk7, 1kzn, 1lee,1 lfv, 1dzj, 1m5u, 1m8s, 1nfg, 1oz4, 3gwx, 1ev5, 1evg, 1f3u, 1g1b, 1p9h, 1r30, 1tve, 1hw3, 1hw4, 1ijb, 1izp, 1izq, 1ktk, 2gwx). Blue diamonds and red squares correspond to the bulk-solvent parameters calculated in *CCTBX* using least-squares (LS) and maximum-likelihood (ML) target functions, respectively. Black triangles represent the bulk-solvent parameters reported in the PDB file under keywords ‘KSOL’ and ‘BSOL’.

In most cases the new procedure yields physically reasonable parameters using both LS and ML target functions (Fig. 3 ▶). However, for some models (for example, PDB codes 1jh7, 1k33, 1kk7, 1lee, 1r30 and 2gwx) the parameters *k*_sol_ and *B*_sol_ were outside the reasonable range, which may indicate insufficient data or poor model quality. In such cases the procedure sets the parameters to the best found in the search grid in step I (Fig. 1 ▶).

**Figure 4:**
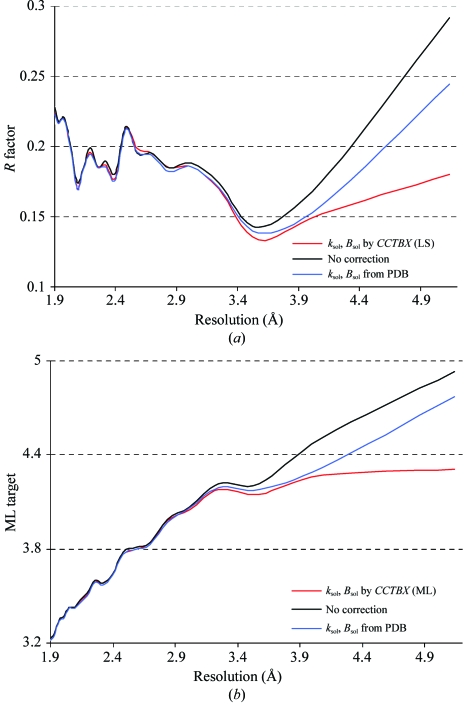
*R* factor (*a*) and ML function (*b*) (ML is normalized by the number of reflections in bins) calculated in resolution bins (for the structure with PDB code 1jj1): no scaling and bulk-solvent correction (black), parameters *k*_sol_ and *B*_sol_ and scale matrix **B**_cart_ taken from the PDB file (blue), scaling and bulk-solvent correction parameters calculated using *CCTBX* with the least-squares (*a*) and maximum-likelihood target (*b*).

In order to evaluate the model improvement arising from more reasonable bulk-solvent parameters, *R* factors *versus* resolution were calculated for all selected models and a typical example for one model (PDB code 1jj1) is presented in Fig. 4 ▶(*a*). The use of corrected parameters significantly improves the fit for the low-resolution data, while the *R* factor calculated with the unreasonable parameters, taken from the PDB file, is 6% higher in the lowest resolution shell and about 11% higher for the case where no correction was performed. Analogous calculations were performed using the maximum-likelihood target function (Fig. 4 ▶ *b*). Again, the parameters determined with the new method improve the likelihood target function compared with calculations with incorrect parameters or without any scaling and solvent correction.

**Figure 5:**
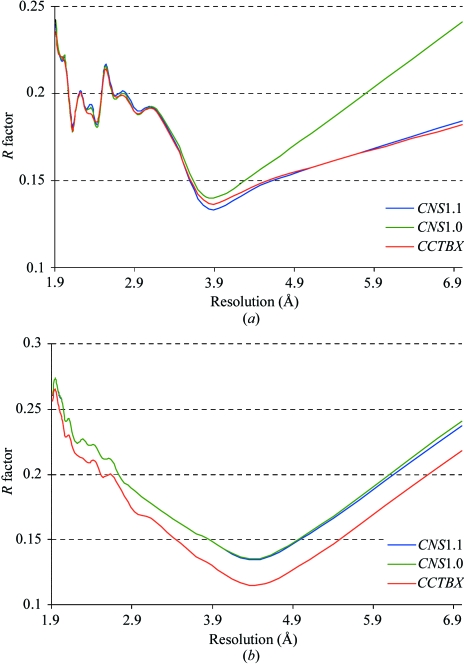
*R* factor as a function of resolution (in Å) for the structures with PDB code 1jj1 (*a*) and 1lee (*b*). Bulk-solvent correction and anisotropic scaling performed with *CNS*1.0 (green), *CNS*1.1 (blue) and *CCTBX* (red).

In addition, tests were performed in order to compare the calculation of flat bulk-solvent and anisotropic scaling parameters in selected programs that provide this option (Fig. 5 ▶). In many cases *CNS*1.1 performs significantly better then *CNS*1.0 (Fig. 5 ▶ *a*). This is because the bulk-solvent correction procedure in *CNS*1.1 was improved by changing the initial values for *k*_sol_ and *B*_sol_ from zero to the observed mean values (Fokine & Urzhumtsev, 2002*a* ▶), 0.35 e Å^−3^ and 46.0 Å^2^, respectively. In some cases *CNS*1.1 gives similar or slightly worse results than *CCTBX* (Fig. 5 ▶ *a*). However, there are cases where the new procedure gives noticeably better results than both *CNS*1.0 and *CNS*1.1 (Fig. 5 ▶ *b*). Finally, analogous calculations of flat bulk-solvent correction and anisotropic scaling with *REFMAC* using the SCALE SIMPLE option gave similar results to those seen with *CNS*1.0.

## 6. Conclusions

A robust method for the determination of anisotropic scale factor and flat bulk-solvent model parameters is required as structure determination becomes more automated. The new method we have described here, in combination with the likelihood function for optimization of the parameters, will minimize the occurrence of errors. The robustness of the algorithm has been proven on 35 structures selected from the PDB where unreasonable bulk-solvent parameters were reported. In most of these cases the new procedure found values close to those typically observed in refined structures. In our tests, the new procedure is as good as or better than *CNS*1.1 or *REFMAC* in determining optimum parameters for typical structures and works significantly better for ‘problem’ structures.

These new algorithms are implemented in the *CCTBX* bulk-solvent correction and scaling module. *CCTBX* is available as open-source software at http://cctbx.sourceforge.net. All results presented are based on the *CCTBX* source code bundle with the version tag 2005_03_02_2358.

## Appendix A The derivatives of maximum-likelihood target function with respect to bulk-solvent parameters and components of the anisotropic scale matrix

Given the function Ψ defined by (6) its derivatives with respect to the six anisotropic scale-matrix elements (*B*_cart_)*_ij_* can be obtained following the chain rule,

where the function  is defined below.

The calculation of derivatives with respect to the bulk-solvent parameters *k*_sol_ and *B*_sol_ requires more attention. We can define a function (**z**) of complex variables as **z** = **u** + *g*(*p*)**v**, where **u** and **v** are complex variables and *g*(*p*) is a function with real arguments. Remembering that |**z**| = (**z*****z**)^1/2^ and using the chain rule, one can obtain the derivative with respect to *p* as

Replacing **u**, **v** and *g*(*p*) with ,  and *k*_sol_exp(−*B*_sol_ *s* ^2^/4), the desired derivatives are

where

and
